# Effects of strength exercise interventions on activities of daily living, motor performance, and physical activity in children and adolescents with leukemia or non-Hodgkin lymphoma: Results from the randomized controlled ActiveADL Study

**DOI:** 10.3389/fped.2022.982996

**Published:** 2022-11-08

**Authors:** Dominik Gaser, Christiane Peters, Renate Oberhoffer-Fritz, Miriam Götte, Tobias Feuchtinger, Irene Schmid, Bernhard Haller, Irene von Luettichau, Sabine Kesting

**Affiliations:** ^1^ Kinderklinik München Schwabing, Department of Pediatrics and Children’s Cancer Research Centre, TUM School of Medicine, Technical University of Munich, Munich, Germany; ^2^ Chair of Preventive Pediatrics, Department of Sport and Health Sciences, Technical University of Munich, Munich, Germany; ^3^ Pediatric Oncology Network, KIONET Bavaria, Erlangen, Germany; ^4^ Clinic of Pediatrics III, Department of Hematology and Oncology, West German Cancer Centre Essen, University Hospital, Essen, Germany; ^5^ Dr. von Hauner Children’s Hospital, Pediatric Hematology and Oncology, Ludwig-Maximilians-University Munich, Munich, Germany; ^6^ Institute of AI and Informatics in Medicine, TUM School of Medicine, Technical University of Munich, Munich, Germany

**Keywords:** supervised exercise program, strength training, physical function, motor skills, accelerometry, childhood cancer, intensive treatment

## Abstract

**Objectives:**

Pediatric patients with cancer experience impairments in muscle strength and physical activity (PA) that may reduce autonomy during hospitalization. To determine the effects of strength exercise interventions on the accomplishment of activities of daily living (ADLs), motor performance, and PA in children with leukemia or non-Hodgkin lymphoma, we randomly allocated patients (4–18 years) immediately after diagnosis into two exercise groups.

**Methods:**

The intervention group (IG; *n *= 21) received a specific strength training combined with a standard care exercise program, whereas the control group (CG; *n *= 20) was provided standard care exercise program without any targeted muscle strengthening. After the baseline visit, participants were followed-up three times until intensive treatment cessation. We assessed physical function limitations using the Activities Scale for Kids© (ASK) and Functional ADL Screen. Secondary outcomes were PA levels using accelerometer and motor performance as measured by MOON-test (motor performance in pediatric oncology-test).

**Results:**

In both groups, ADL accomplishment had significantly increased (*p* < 0.05). However, no significant between-group differences for ASK outcome were noted. Motor performance was reduced in all motor abilities.

**Conclusions:**

Both exercise interventions were effective to maintain ADLs and motor performance during intensive treatment. In comparison, regular strength exercise interventions in the course of therapy tended to be more beneficial with regards to muscular explosive and endurance strength.

## Introduction

Regular exercise for children with cancer is strongly recommended by international guidelines (1, 2). However, professional exercise programs are not implemented nationwide in pediatric oncology departments so far in Germany (3) and are rarely distributed worldwide (4). Disease- and treatment-related implications throughout intensive treatment can cause restrictions in the patients' activity level (5), which can be further associated with decline in cardiorespiratory fitness (6) and motor performance (7). Patients with leukemia reduce their daily step counts by 70% compared with healthy peers during inpatient stays (8). As a consequence, the amount of physical activity (PA) is reduced through treatment by up to 91% (9). In a large number of patients, impairments persist well beyond therapy cessation and into adulthood (10–12). Recently, the number of published studies increased regarding exercise intervention among pediatric patients with cancer and consequently provided growing evidence concerning PA during intensive treatment (13). Thus, positive effects of exercise interventions on exercise capacity (14), cardiorespiratory fitness (15), fatigue (16), muscle strength (17), and PA levels (18) have been shown in pediatric cohorts with mixed cancer entities. Furthermore, data increasingly identified the potential of PA and exercise during follow-up care to reduce disease- and therapy-related late effects, including fatigue (19), obesity (20), or cardiovascular diseases (21), as well as all-cause mortality among childhood cancer survivors (22).

Strength ability assumes a central role in general locomotion and the execution of everyday tasks. Muscle strength is needed for all levels of physical activity. In older adults, low muscle mass and reduced muscle strength are associated with activities of daily living (ADLs) dependency (23). Reduced strength ability is evident in children with acute lymphoblastic leukemia (ALL) during acute therapy (24). As previously described, patients with other pediatric diseases showed reduced muscle strength, which may have an impact on their autonomous coping with ADLs (25, 26). However, little is known about the functional impairments affecting ADLs induced by cancer and the therapy duration, or about potential benefits of strength training for ADL accomplishment throughout the intensive treatment period. From a clinical perspective, ADL impairments typically become obvious as an additional burden upon the disease for these patients, especially for adolescents, considering the need for assistance and dependency on parents or caregivers. Therefore, even essential human needs (e.g., getting up, putting on clothes, using the toilet) can become insurmountable hurdles in clinical routine and may affect children's autonomy and mobility. In our recent publication, patients have shown multifunctional impairments in self-reported ADLs immediately after the diagnosis of leukemia or non-Hodgkin lymphoma (NHL) (27). Furthermore, long-term childhood cancer survivors experienced limitations in physical function (28) and ADL accomplishment, including personal care, routine activities, or attending work, compared with their siblings (29). Accordingly, tailored exercise interventions during intensive treatment could promote children's autonomy and strengthen both physical and mental well-being.

This randomized controlled trial (RCT) aimed to determine the effects of regular supervised strength exercise interventions on self-reported ADLs, motor performance, and PA among pediatric patients with cancer with ALL or acute myeloid leukemia (AML) or NHL during acute treatment. We hypothesized that a specific strength training would be a more appropriate method than our standard care exercise program without targeted strengthening interventions to improve the primary outcome of ADL accomplishment until intensive treatment cessation. Secondary outcomes were exercise effects on motor performance (including functional strength, speed, coordination, flexibility) and PA levels in the course of treatment.

## Materials and methods

### Participants and study design

The exploratory bicentric ActiveADL Study (ClinicalTrials.gov: NCT03934060) followed a randomized controlled design in adherence to the ethical guidelines of the Declaration of Helsinki. This study was approved by the Ethics Committees of TUM School of Medicine, Technical University of Munich (TUM; 25/17 S) and University of Munich (18-323). The study content was communicated orally and in written form to the eligible patients. The children's legal guardians and all participants aged ≥16 years provided written informed consent to participate in this study. The ActiveADL Study was conducted at the Children's Hospital Schwabing and Dr. von Hauner Children's Hospital in Munich between September 2017 and February 2021 (last patient-in: June 2020). Eligibility criteria included those aged 4–18 years, with primary or secondary diagnosis (5 years post-primary tumor) of ALL, AML, or NHL. The exclusion criteria were as follows: patients with medical contraindications for PA post-diagnosis (i.e., thrombosis, high risk of bleedings, or fractures), those with the absence of German or English language abilities, and those who communicated a change of hospital during the first weeks of treatment. The lower age limit was selected to realize the exercise methods, particularly strength training for children, and allow the comparison of outcome measures with normative data, respectively. Allocation to the control (CG) and intervention group (IG) was based on a predefined block randomization schedule (with a block size of four).

### Sample size

Prior to the ActiveADL Study, power analysis to estimate the intervention effect was not feasible due to the lack of reliable data from previous studies. All patients who met the inclusion criteria were asked to participate since the case numbers are small *per se* owing to the low incidences in pediatric oncology. Considering the potential initial diagnosis at both study sites, the case number estimation over a 2-year recruitment period resulted in a total number of 20 participants in the IG and CG, respectively. A possible 10% dropout (participation decline or death) was considered.

### Exercise interventions

All participants followed an in-hospital tailored exercise program that occurred during the entire acute treatment period. The program included 2–3 exercise sessions per week. All exercise sessions were supervised and documented by exercise physiologists; the training load was oriented toward the participants' physical capacity, current health status, and age. Participants individually performed the exercise sessions inside their rooms, on the corridor, or outdoor in the hospital area. Despite isolation and contact reduction during the COVID-19 pandemic (05/2020–01/2021), exercise intervention continuation was constantly ensured. To ensure the amount of training during this period, supervised web-based exercise sessions were additionally offered using the video conferencing platform Zoom (Zoom Video Communications, Inc., San Jose, CA, United States), if necessary. Before each session, the physiologists screened the participants' general health condition together with physicians. The following were the potential contraindications for exercise: fever combined with fatigue or vertigo, vomiting, diarrhea, lumbar puncture procedure, pneumonia, sepsis, severe pain, and intensive care periods. The IG received a specific strength training combined with a standard care exercise program, whereas the CG was provided standard care without any targeted muscle strengthening to the same extent. Standard care exercise program included sportive games, aerobic or coordination exercises. Group-specific contents of exercise interventions are outlined in Table 1. Due to the heterogeneous cohort according to common age distribution in pediatric oncology, two strength training modules were developed and adapted to the age of the participants (4–8 and 9–18 years). Both modules contained four identical exercise emphases with 40–45 child-friendly and playful exercises on core stability, complex full-body strengthening, and upper and lower body strengthening. Each specific strength training consisted of four exercises and included one exercise from each emphasis. For each exercise, 2–3 sets were performed with a 60 s rest period between sets and a 90 s break between exercises. With the selection of exercises, all body regions and large muscle groups were explicitly trained. Additional warm-up and cool-down exercises aimed to achieve the 30-min training duration. Exercise intensity was not defined on the basis of physiological parameters—for example, one-repetition maximum for resistance exercise or heart rate peak for aerobic exercise—although was determined and increased individually depending on the physiologists' discretion. The IG focused on strength endurance exercise. According to hygiene standards, solely disinfectable and mobile training devices—including kettlebells/dumbbells, swinging bars, resistance bands, balance pads/boards, bicycle ergometers, or aerobic steppers—were used. Additionally, the IG exercised against the resistance of their body weight. Exercise interventions of both groups started after baseline visit immediately after diagnosis. All participants had access to standard care physiotherapy during the intensive treatment. Particularly, participants with severe functional limitations received regular physiotherapy. Physiotherapeutic measures did not provide targeted exercise interventions; however, the measures included were as follows: respiratory therapy, massages for pain relief, mobilization after catheter surgery or intensive care treatment, muscle stretching, medical brace fitting, lymphatic drainage, and fall prevention.

**Table 1:** Contents of exercise interventions by group.

	Intervention group	Control group
Training method	Specific strength training combined with standard care exercise program	Standard care exercise program
Training emphases	Strength AND Endurance/coordination Sportive games Flexibility/relaxation	Endurance/coordination Sportive games Flexibility/relaxation
**Exercise session example**		
Warm-up (5 min)	Bicycle ergometer, joint mobilization	Walking
Main part (20 min)	Elbow planks, squats combined with shoulder press, biceps curls, sidewalks with resistance band	Coordination with juggling balls, table tennis
Cool-down (5 min)	Muscle relaxation	Fantasy relaxation

Min, minutes.

The amount of training for both groups was identical with 30 min per exercise session and 2–3 sessions per week.

### Data collection and assessments

All participants were assessed at four inpatient visits: at baseline (V0), after the induction phase (V1), after the consolidation phase (V2), and at intensive treatment cessation before the transition to maintenance therapy or follow-up care (V3). A combination of participation/activity-based and impairment-based measures was used to evaluate functional limitations of ADL accomplishment and individual motor skills: ADL accomplishment was investigated using the Activities Scale for Kids© (ASK), performance version (ASKp), a self-report measure of childhood physical function (30). Furthermore, the self-developed Functional ADL Screen was used to objectively verify functional limitations in ADL performance (27). Motor performance (including functional strength, speed, coordination, flexibility) was assessed using the MOON-test (motor performance in pediatric oncology), a standardized motor performance diagnostic tool in clinical routine among pediatric patients with cancer (31). Functional motor performance measures are feasible in pediatric oncology (32, 33). Compared to isolated strength testing, functional measures provide immediate conclusions regarding the ability to perform everyday tasks. Based on the testing results, the exercise interventions were controlled and adjusted. PA was measured using the accelerometer Move 3 (movisens GmbH, Karlsruhe, Germany) at outpatient periods for seven consecutive days. While the primary outcome—ADL accomplishment based on the ASK and Functional ADL Screen—was collected at all visits, secondary outcomes—MOON-test and PA—were conducted at V0 and V2. Medical data were collected from patients' records. Anthropometric data were measured using the scale seca 701 and stadiometer seca 216 (seca GmbH & Co., Hamburg, Germany). We calculated the PA level, step count, and wear time using the DataAnalyser software (version 1.13.16). The exercise physiologists documented participants' adherence to the exercise sessions. Adverse events concerning exercise interventions or assessments were recorded with regard to the study of Gauß et al. (34). For a detailed description of the measurement methods, we refer to the publication of the baseline data (27).

### Statistical analysis

Participants with valid recordings of at least 8 h/day for 4 days were included in the analysis. For all outcomes, between-group differences were assessed using two-sample *t* tests (normally distributed data) or Mann–Whitney *U* tests for non-normally distributed data. Longitudinal within-group differences between visits were assessed using paired *t* tests or the Wilcoxon signed-rank tests. For the MOON-test, comparisons with a healthy population were made using age- and gender-matched reference values. Differences from these reference values were analyzed using one-sample *t* test or the Wilcoxon signed-rank test as a non-parametric alternative. Statistical analysis was performed using SPSS version 25. Graphs were created using GraphPad Prism version 9. Data analyses were performed following the intention-to-treat principle; for one participant who did not complete the study data, prior elimination was included. Data are presented as mean ± standard deviation for continuous variables and as absolute and relative frequencies for categorical variables. For continuous data, median and range are also presented to assess the skewness of the distribution. Due to the exploratory nature of the study, a level of significance of *α* = 0.10 was used and 90% confidence intervals were estimated. All statistical tests were performed two-sided.

## Results

### Participant recruitment and visits

**Figure 1:**
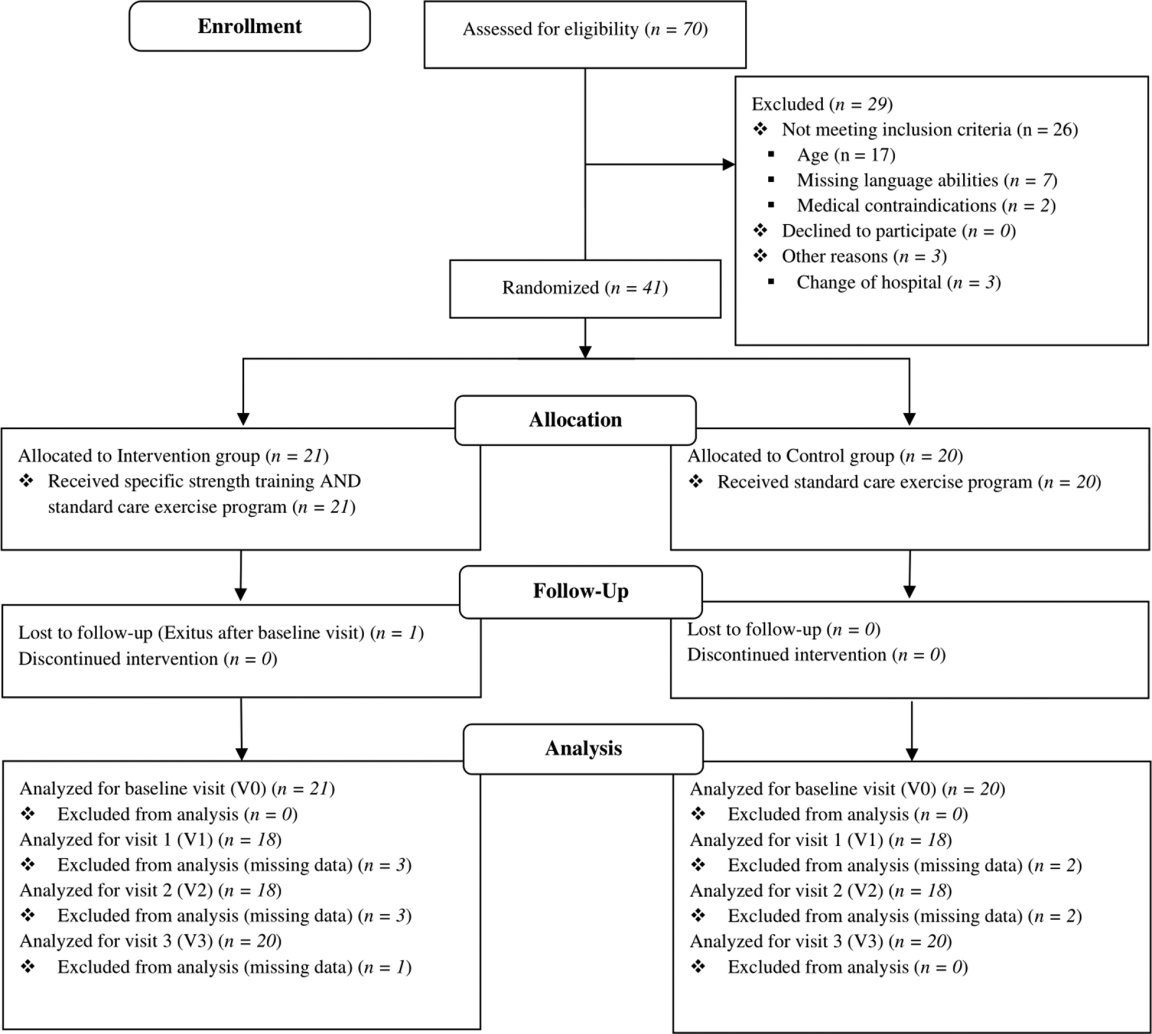
Participant flow following CONSORT 2010 (35). Medical contraindications were pneumonia/sepsis with long-term ventilation and intussusception with biliary colic.

A flow diagram of the study participants is shown in Figure 1. From September 2017, a total of 70 children were screened eligible. Of them, 41 met all inclusion criteria and entered the study until June 2020. They were randomized in the IG (*n *= 21) or CG (*n *= 20). All participants completed the baseline assessment at V0. To achieve the recruitment goal, a 41st participant was included because one participant of the IG died after V0 3.3 months after recruitment without completing the follow-up visits. Thirty-six patients performed the assessments at V1 and V2 due to the different treatment protocols and individual capacity during the course of treatment. The final assessment (V3) was completed by 40 participants.

Participants’ characteristics, including disease- and treatment-related information divided by group, are shown in Table 2. Male participants were in the majority in both groups. The age distribution was nearly balanced. Overall, participants with ALL (*n *= 25) were the predominant number. One participant in the IG underwent allogeneic stem cell transplantation 104 days after V1. Four participants were fitted with a brace owing to vertebral compression fractures. The brace was worn during the exercise interventions and assessments.

**Table 2:** Participants’ anthropometric and clinical baseline characteristics by group.

Characteristics	Intervention group (*n* = 21)				Control group (*n* = 20)			
	*N* (%)	M ± SD	Median	Range	*N* (%)	M ± SD	Median	Range
**Recruitment**								
Age at recruitment (years)	21 (100)	10.2 ± 4.2	10.1	4.4–17.1	20 (100)	9.7 ± 3.9	9.2	4.3–17.5
Days post-diagnosis	21 (100)	14.7 ± 9.1	13.0	3–49	20 (100)	15.6 ± 11.4	11.5	4–52
**Gender and age (years)**								
Male	15 (71)	10.4 ± 4.0	10.8	4.4–16.1	12 (60)	9.6 ± 3.8	9.2	5.2–17.5
Female	6 (29)	9.6 ± 5.0	7.2	4.4–17.1	8 (40)	9.9 ± 4.2	9.7	4.3–15.7
BMI (kg/m^2^)	21 (100)	17.2 ± 3.3	15.8	13.3–23.9	20 (100)	17.0 ± 4.8	16.0	12.1–35.1
BMI z-score^a^	21 (100)	−0.3 ± 0.9	−0.3	−2.2 to 1.4	20 (100)	0.5 ± 1.5	−0.4	−3.5 to 3.1
**Tumor type and age (years)**								
ALL	11 (52)	9.5 ± 4.6	8.5	4.4–17.1	14 (70)	8.5 ± 3.3	8.0	4.3–15.7
AML	1 (5)	16.1	16.1	16.1	3 (15)	12.4 ± 2.5	11.1	10.8–15.3
NHL	9 (43)	10.3 ± 3.5	12.0	6.1–15.4	3 (15)	13.1 ± 5.2	14.5	7.3–17.5
Second primary cancer^b^	1 (5)				1 (5)			
**Treatment** ^c^								
Chemotherapy	21 (100)				20 (100)			
Radiation therapy	1 (5)				2 (10)			
Allogeneic HSCT	1 (5)				0 (0)			
Medical brace^d^	0 (0)				4 (20)			

ALL, acute lymphoblastic leukemia; AML, acute myeloid leukemia; HSCT, hematopoietic stem cell transplantation; NHL, non-Hodgkin lymphoma; M, mean; SD, standard deviation; N, number; BMI, body mass index; kg, kilogram; m^2^, square meter; mg, milligram. Gender-, age-, and disease-related information were determined from hospital records.

^a^ BMI z-score was calculated using gender- and age-adjusted reference values (36).

^b^ Participants who were diagnosed with a second primary cancer >5 years after the first treatment: *n* = 1 ALL → NHL after 15 years; *n* = 1 NHL → different NHL type after 8 years. The participants had completely recovered and had no limitations or long-term effects of the primary tumor.

^c^ Characteristics on treatment methods refer to the entire study course.

^d^ Medical brace in the course of treatment was necessary in cases of osteoporotic vertebral compression fractures in four participants. This limited the upper body mobility during the assessment. The exercise intervention implementation was not restricted.

Intervals between study inclusion and each visit are presented in Table 3. Assessments of the two groups were performed at similar measurement points during treatment.

**Table 3:** Intervals between study recruitment and follow-up visits by group.

Characteristics	Intervention group				Control group			
	*N*	M ± SD	Median	Range	*N*	M ± SD	Median	Range
From recruitment to V0 (days)	21	4.9 ± 9.0	1.0	0–36	20	3.2 ± 3.8	1.5	0–14
From recruitment to V1 (days)	18	65.7 ± 25.3	62.0	26–106	18	60.2 ± 15.6	60.0	38–98
From recruitment to V2 (days)	18	141.7 ± 47.0	141.0	53–216	18	143.3 ± 35.9	142.5	57–190
From recruitment to V3 (days)	20	223.7 ± 92.4	224.5	76–449	20	235.0 ± 44.9	247.5	120–322

M, mean; N, number; SD, standard deviation; V, visit.

### Exercise interventions, adverse events, and adherence

Exercise characteristics are shown in Table 4. Over a mean exercise intervention period of 7.1 ± 3.1 months, the IG completed 33.0 ± 20.0 exercise sessions (range, 10–84 sessions). In comparison, the CG performed 40.9 ± 20.6 sessions (range, 15–79 sessions) over a period of 7.7 ± 1.5 months. The IG rejected an average of 18.1 ± 12.8 exercise sessions over the intervention period compared with 18.9 ± 9.4 sessions in the CG. Adherence to the exercise sessions throughout the intervention period in relation to all exercise sessions offered was 65% and 68% in the IG and CG, respectively. Among all participants, the average exercise session duration was 29.7 ± 4.7 (range, 22–44) min. No serious adverse events (grades 2–5) or injuries associated with 1,539 exercise interventions, including 29 supervised web-based sessions (distributed among five participants of the IG) and 153 assessments, were recorded. One minor treatment-related event (vomiting) led to exercise session termination. One participant stumbled during balance training without any consequences and was able to continue the exercise session. For another participant, the MOON-test was discontinued due to treatment-related nausea and vertigo and continued the following day. No participants were lost to follow-up due to personal or exercise-related reasons.

**Table 4:** Exercise characteristics by group.

Characteristics	Intervention group (*n* = 21)			Control group (*n *= 20)		
	M ± SD	Median	Range	M ± SD	Median	Range
Total exercise intervention duration (months)	7.1 ± 3.1	7.3	2.5–14.7	7.7 ± 1.5	8.1	3.9–10.6
Exercise sessions	33.0 ± 20.0	28.0	10–84	40.9 ± 20.6	37.5	15.0–79.0
Exercise sessions rejected	18.1 ± 12.8	13.0	2–57	18.9 ± 9.4	16.5	5.0–39.0
Mean number of exercise sessions/week	1.0 ± 0.4	1.1	0.4–2.1	1.2 ± 0.5	1.2	0.5–2.2
Mean number of potential exercise sessions/week^a^	1.6 ± 0.6	1.6	0.6–2.7	1.8 ± 0.6	2.0	1.0–2.8
Mean exercise session duration (min)	30.6 ± 4.2	30.0	23.5–42.0	28.9 ± 5.2	27.5	22.2–43.6

M, mean; min, minutes; *N*, number; SD, standard deviation.

^a^ The quotient of the number of completed exercise sessions and the total number of potential exercise sessions, considering the rejected sessions.

### Activities of daily living

**Figure 2:**
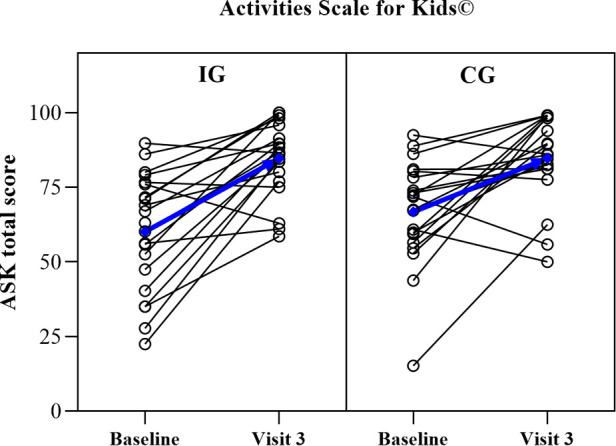
Within-participant development from the baseline (Visit 0) to Visit 3 for the activities scale for Kids© total score in the intervention (IG, *n* = 20) and control group (CG, *n *= 20). Blue lines represent within-group mean development.

The analysis of self-reported ADLs (Table 5) revealed no between-group differences at V3. The CG significantly increased the ASK total score (18.0 ± 20.5; *p *< 0.001) in the course of treatment (V0–V3). Within-group analysis of the IG revealed a significant ASK total score improvement (24.7 ± 20.3; *p *< 0.001) between V0 and V3 (Figure 2). The analysis of the Functional ADL Screen showed an improvement of the total score from V0 to V3 in the IG compared with the CG (1.9 ± 4.4; *p* = 0.034 vs. −0.1 ± 5.4; *p* = 0.916). In the IG, seven participants required locomotion support indoor and/or outdoor in the study course, compared with eight participants in the CG. Reported aids for locomotion were wheelchairs, crutches, hands and knees, and carried by parents.

**Table 5:** Effects of exercise interventions on activities of daily living by group and visits.

Outcome	Group	M ± SD (90% CI); *N* at V0	*Δ* from V0 to V1 (90% CI); *N*	*Δ* from V0 to V2 (90% CI); *N*	M ± SD (90% CI); *N* at V3	*P* within groups (V0–V3)	*P* between groups at V3
**Self-reported ADL**							
ASKp score^a,b^ (range, 0–100)	CG	69.4 ± 19.8 (60–79); 14	8.7 (−2 to 20); 12	13.2 (3–23); 12	86.7 ± 12.2 (74–86); 19	0.005	0.822
	IG	62.6 ± 18.8 (52–73); 11	8.0 (−6 to 22); 9	17.8 (10–31); 10	84.7 ± 12.5 (80–90); 20	0.010	
ASK total score^b^ (range, 0–100)	CG	66.8 ± 17.7 (60–74); 20	9.1 (0–18); 18	14.7 (6–23); 18	84.8 ± 14.4 (79–90); 20	<0.001	0.978
	IG	60.1 ± 19.5 (53–67); 21	20.1 (10–30); 18	23.2 (16–30); 18	84.7 ± 12.5 (80–90); 20	<0.001	
**Functional ADL Screen**							
Total score^c^ (range, 0–28)	CG	27.0 ± 3.7 (25–28); 19	0 (−2 to 2); 17	−0.6 (−3 to 2); 17	26.9 ± 3.8 (25–28); 20	0.916	0.043
	IG	26.1 ± 4.2 (25–28); 21	0.8 (−1 to 3); 18	1.0 (0–2); 17	28.0 ± 0.2 (28); 19	0.034	

ASK/ASKp, Activities Scale for Kids©/Activities Scale for Kids© performance version; CI, confidence interval of differences; CG, control group; IG, intervention group; M, mean; *N*, number; *P*, *p*-value; SD, standard deviation; V, visit.

Score changes (mean) from the baseline (V0) to V3 within the groups were assessed using paired *t* tests (ASKp/ASK total score in the IG; ASK total score in the CG), and Wilcoxon singed-rank tests for all other longitudinal comparisons, respectively. Between-group differences were assessed using two-sample *t* tests for ASK total score or Mann–Whitney *U* tests for ASKp score and Functional ADL Screen. Positive *Δ*-values represent score improvements.

^a^ Case number differences between ASKp score and ASK total score based on the different calculation methods. The prerequisite to calculate the ASKp score was 23 out of 30 valid responses (30).

^b^ A total score of 100 indicates no functional limitations.

^c^ A total score of 28 indicates no functional limitations.

### Motor performance

The MOON-test results are presented in Table 6. The number of tested participants varies within test items due to published age-specific reference values and limited individual capacity. Except for muscular explosive strength at V2 (IG: −20.3 ± 8.0 vs. CG: −34.5 ± 12.8; *p* = 0.012), no significant between-group differences were noted. In the IG, we observed improved mean values for eye-hand coordination (*p *= 0.177), static balance (*p *= 0.325), speed (*p *= 0.016), muscular explosive strength (*p *= 0.214), and muscular endurance for the legs (*p *= 0.011) at V2 than at V0. Moreover, in the CG, mean value changes in eye-hand coordination (*p *= 0.983), static balance (*p *= 0.221), speed (*p *= 0.158), upper extremity coordination (*p *= 0.465), and muscular endurance for the legs (*p *= 0.407) were observed between V0 and V2. The intergroup comparison of means provided results that were below the age- and gender-matched reference values in almost all eight motor abilities.

**Table 6:** Effects of exercise interventions on motor performance by group and visits.

				Intervention group					Control group					
				* *	*Differences to reference values (%)*					*Differences to reference values (%)*				
Motor ability	Test item		Visit	*N*	M ± SD	Median	90% CI	Scoring below reference (%)	*N*	M ± SD	Median	90% CI	Scoring below reference (%)	*P* between groups
Eye-hand coordination	Inserting pins (time in s)		V0	21	−15.3 ± 32.3	−6.9	(−27 to −3)	71	20	−13.7 ± 30.6	−10.4	(−26 to −2)	70	0.896
			V2	17	−5.1 ± 32.9	−1.6	(−19 to 9)	53	18	−12.2 ± 34.0	−2.2	(−26 to 2)	50	0.947
Static balance	Static stand (*n* of contacts)^b,c^		V0	19	15.4 ± 65.3	−2.0	(−11 to 41)	42	15	1.3 ± 6.7	0.3	(−2 to 4)	60	0.835
			V2	17	0.4 ± 6.9	−3.7	(−2 to 3)	41	14	0 ± 6.7	−2.8	(−3 to 3)	29	0.736
Speed	Reaction test (time in s)		V0	20	−12.9 ± 14.9	−13.9	(−19 to −7)	80	19	−15.4 ± 31.8	−8.3	(−28 to −3)	68	0.536
			V2	17	−4.1 ± 16.2	0	(−11 to 3)	47	18	−10.5 ± 22.1	−5.7	(−20 to −1)	56	0.498
Upper extremity coordination	Throwing at a target (hits)^a^		V0	8	−16.3 ± 76.2	−43.6	(−67 to 35)	63	8	−16.8 ± 30.1	−23.8	(−37 to 3)	63	0.505
			V2	6	−35.6 ± 17.6	−35.8	(−50 to −21)	100	5	−9.6 ± 35.1	0	(−43 to 24)	40	0.329
Flexibility	Stand-and-reach (difference in cm)^b^		V0	19	−9.2 ± 11.4	−6.0	(−14 to −5)	79	20	−9.2 ± 12.0	−6.8	(−14 to −5)	65	0.736
			V2	17	−9.8 ± 11.3	−10.4	(−14 to −5)	82	18	−10.2 ± 10.3	−11.8	(−14 to −6)	83	0.717
Muscular explosive strength	Medicine ball shot (distance in m)^a^		V0	14	−23.4 ± 16.0	−19.5	(−31 to −16)	100	11	−27.2 ± 18.2	−29.5	(−37 to −17)	91	0.647
			V2	14	−20.3 ± 8.0	−17.3	(−29 to −12)	86	13	−34.5 ± 12.8	−35.7	(−41 to −28)	100	0.012
Muscular endurance for the legs	Sit-to-stand (time in s)		V0	20	−46.1 ± 100.0	−25.1	(−82 to −10)	90	20	−24.9 ± 37.0	−16.7	(−39 to −11)	65	0.607
			V2	17	−18.0 ± 66.6	5.2	(−46 to 10)	35	17	−11.4 ± 26.1	−1.3	(−22 to 0)	59	0.547
Hand grip strength	Hand-held dynamometry (strength in kg)^a^	Right	V0	17	−20.3 ± 24.6	−23.2	(−31 to −10)	82	16	−29.5 ± 16.2	−27.3	(−37 to −22)	94	0.296
			V2	15	−22.7 ± 31.5	−27.3	(−37 to −8)	80	13	−32.4 ± 24.8	−32.1	(−39 to −26)	92	0.650
		Left	V0	17	−19.3 ± 26.9	−23.2	(−31 to −8)	77	16	−26.4 ± 18.1	−23.3	(−34 to −18)	100	0.601
			V2	15	−21.3 ± 29.8	−26.4	(−35 to −8)	80	14	−30.4 ± 15.8	−30.7	(−38 to −23)	93	0.652

CI, confidence interval; cm, centimeter; kg, kilogram; m, meter; M, mean; *N*, number; *P*, *p*-value; s, seconds; SD, standard deviation; V, visit.

sit-to-stand results were compared with unpublished age- and gender-specific pupils reference values (*n* = 289) in Munich (37). All other results were compared with the reference values of each single test items (31, 38). Single test items could not be assessed or analyzed due to missing reference values or restricted health status.

^a^ Because of published reference values, throwing at a target is limited to participants between 6 and 11 years of age, the test items medicine ball shot and hand-held dynamometry are limited between 6 and 18 years of age.

^b^ Absolute differences to reference values were calculated, because values around zero lead to exaggerated percentage values.

^c^ A positive difference in static stand represents a result below the reference values, because it means a higher number of ground contacts of the free leg.

### Physical activity

**Figure 3:**
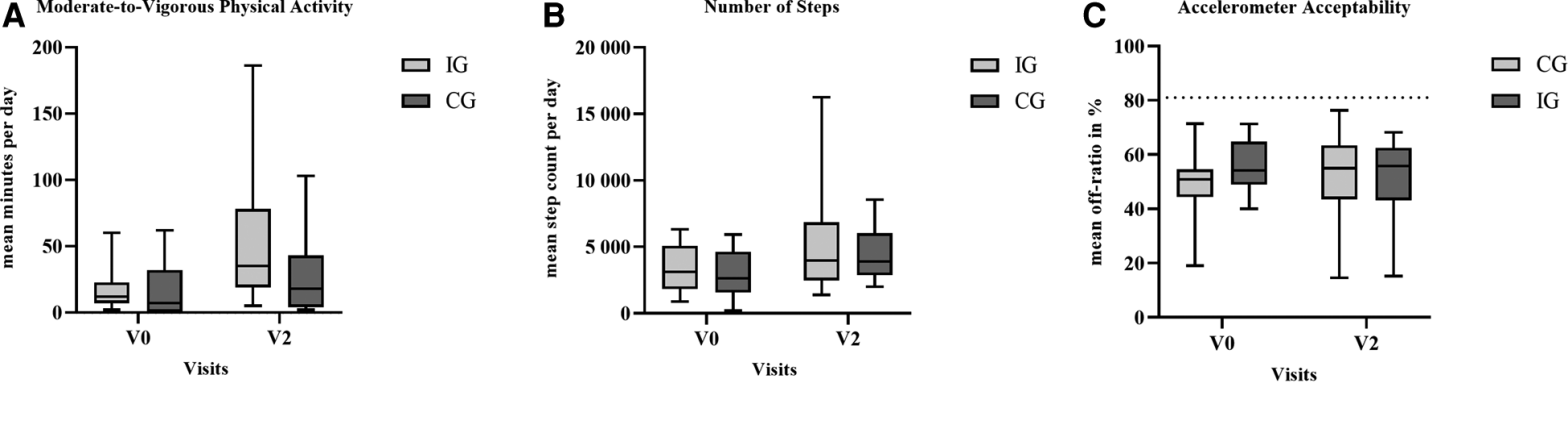
Between-group differences at the baseline (V0) and Visit 2 regarding the outcome (**A**) moderate-to-vigorous physical activity; (**B**) physical activity amplitude, and (**C**) accelerometer acceptability. The dashed line at 81% off-ratio represents the minimum accelerometer wearing time of 4 days with 8 h/day. Note: Whiskers represent the minimum and maximum of the cases.

Overall, 72 accelerometer measurements were conducted across both groups, including 61 valid recordings at V0 and V2. The mean wear time of valid recordings was 5.9 ± 1.0 days (maximum of 7 days). Reasons for invalid measurements were the lack of compliance and unscheduled inpatient hospitalizations. Both the CG (V0: 17 ± 21 min, range 0–102 min, *n* = 13; V2: 25 ± 29 min, range 2–103 min, *n* = 11) and IG (V0: 18 ± 18 min, range 2–60 min, *n* = 13; V2: 49 ± 51 min, range 5–186 min, *n* = 11) increased their level of moderate-to-vigorous PA (MVPA, ≥3 METs) in the course of therapy (Figure 3). The mean number of steps per day increased between V0 and V2 within CG (+37%) and IG (+44%). At V2, five participants from the IG achieved a step count of >10,000 steps on individual days throughout the investigated week, whereas two participants from the control group achieved >10,000 steps. In the IG, the accelerometer acceptability, presented as mean relative off-ratio, was 56% at V0, and 52% at V2, respectively, compared with the CG (49% at V0; 52% at V2). No significant between-group differences were observed.

## Discussion

To our knowledge, the ActiveADL Study is the first randomized controlled clinical trial investigating the effects of a specific strength training on the ADL accomplishment in a pediatric cancer cohort during intensive treatment. Only a few studies among pediatric patients with cancer included regular tailored in-hospital exercise interventions throughout the entire acute therapy period (21, 39). Thus, our study provides further insights into the effects of supervised exercise interventions, which are still limited according to published literature due to moderate evidence, study bias or small sample sizes (40, 41). Nevertheless, the potential benefits of exercise on psychosocial and physical parameters have been shown in various intervention studies among children with cancer in recent years (13, 39, 42). Positive effects of exercise interventions during pediatric cancer treatment have been demonstrated regarding increased functional mobility (40), muscle strength (16), health-related quality of life (43), and decreased cancer-related fatigue level (44). Childhood cancer survivors and their parents also reported the importance of being physically active during hospitalization through a combined intervention of physical and social activities and with the motivation of peers (45). However, it is not clearly investigated from which training content patients benefit the most to ensure functionality and autonomy in everyday acute therapy. In particular, for the intensive treatment phase, advantages of supervised exercise interventions over non-supervised independently conducted training protocols became apparent (46). As a minimum level of strength is the basic requirement for locomotion, infantile play and the accomplishment of everyday tasks, the IG received a specific training to maintain their strength abilities. To present differences between both groups as transparently as possible, the CG did not receive any specific exercises to target strength abilities. To answer the research question regarding ADL accomplishment, participants experienced changes in daily living skills and functional impairments were intentionally assessed both, subjectively with the Functional ADL Screen and objectively with the ASK.

The longitudinal analysis has shown that the ASK total score of both groups increased during the course of therapy and only slightly differed from healthy children (mean age, 11.0 ± 2.9 years; *n *= 209; IG: −4.4%; CG: −2.4%) at the end of the intensive treatment (47). Compared with a cohort of patients with bone tumors (*n* = 21, mean 14.9 years of age, mean 2.1 years after tumor surgery), our intervention groups achieved similar ASKp scores at the intensive treatment cessation (48). The data suggest that a specific strength training does not consistently increase the ability to perform better ADL accomplishment. The results of a study in a population of elderly people showed that sarcopenia, defined by physical performance, muscle mass and muscle strength, is associated with impairment of higher-level functional capacity in daily living (49). In a cohort of adult cancer patients, a combined in-hospital physical therapy with resistance, aerobic, and stretching exercises improved muscle strength and functional independence regarding ADLs (50). Also a retrospective analysis among pediatric cancer patients identified that functional limitations in ADLs could be reduced through an inpatient rehabilitation program, including strength training during treatment (51).

Both immediately after diagnosis and after the consolidation phase, our cohort shows impairments in all dimensions of motor performance compared with healthy children and adolescents. These results confirm the findings of a pediatric study population with different cancer entities at the end of acute therapy (7) and those of a cohort of patients with leukemia and lymphoma in the maintenance therapy and follow-up care, respectively (52). The MOON-test results suggest motor skills preservation in both groups in the course of treatment. In comparison, the strength training intervention seems to be slightly superior with regards to muscular explosive and endurance strength.

The accelerometer data show limited PA during outpatient treatment periods in almost all participants. The WHO PA recommendations for children and adolescents of at least 60 min of daily MVPA are only met by a few participants (53). However, our cohort was similarly physically active compared with their healthy peers (54). Both groups increased the amount of PA over the course of the study.

The data on adherence illustrate that, in principle, both—sessions of general exercise promotion and specific strength training interventions—can be implemented throughout the acute therapy period. Based on published exercise studies in pediatric oncology, exercise content, frequency, duration, and adverse events were standardized documented in the ActiveADL Study. Regarding study enrollment, none of the screened 70 patients had to be excluded due to the absence of personal/parental consent. The most common reason for exclusion was the patients' age. This mainly includes children with ALL aged <4 years old. None of the participants prematurely terminated the intervention on their personal initiative, underlining the fact that potentially occurring physical limitations in the course of treatment are manageable by training adjustment.

Our interventions proofed safe and feasible in all age groups (55). No adverse events with consequences that occurred during supervised exercise sessions have been reported. Three Grade 1 adverse events among our participants represent a 0.002% relative frequency. In Germany, fewer adverse events occurred in our cohort than those with other acute cancer clinics with an exercise program (34). After consultation with physicians and based on published literature, one participant continued to exercise after stem cell transplantation and successfully completed the intervention (56, 57).

Due to the lack of practicability, exercise control *via* physiological parameters was dispensed with. Accordingly, following the recommendation of Coombs et al. (39), the training content, dose, and intensity were adapted to the individual's state of health and adjusted to the therapy phase (i.e., outpatient or inpatient) and the children's interests. Furthermore, therapy-related performance changes could be considered, making it possible to provide training stimuli even for participants with low exercise capacity (i.e., sitting or lying in bed). With weekly 1.7 ± 0.6 (range, 0.6–2.8) training opportunities in the cohort, not all participants achieved the intended number of 2–3 exercise sessions per week. Deviations in exercise frequency or duration in relation to the defined training parameters have also been described in other RCTs with shorter intervention periods (17, 56). During the mean intergroup intervention period of 7.4 ± 2.4 (range, 2.5–14.7) months, several factors—for example, therapy-related side effects, limited physical capacity, or outpatient treatment phases—have influenced the exercise frequency. Reasons for exercise session rejections included sobriety prior to invasive procedures, time constraints in clinical routine, and, in rare cases, a lack of motivation. The higher number of exercise sessions in the CG compared with the IG (40.9 ± 20.6 vs. 33.0 ± 20.0 sessions) could be related to the intervention period that was on average 3.5 weeks longer. Variations in treatment regimen led to individual variability in the number of exercise sessions. In conclusion, 2–3 weekly exercise sessions during the intensive treatment are a realistic goal, which should be re-evaluated and, if necessary, adjusted weekly due to the different courses of the disease and individual activity levels.

Considering the findings of our baseline analysis that children and adolescents revealed multifunctional impairments in ADLs, PA and motor performance immediately after diagnosis of ALL, AML or NHL, patients may benefit from early exercise interventions immediately after diagnosis to reduce ADL impairments and maintain motor performance and activity levels until treatment cessation (27). Consequently, the exercise behavior of patients could be positively influenced in the long term to improve coping skills for everyday tasks even in the follow-up care in order to decrease the risk of physical performance limitations among childhood cancer survivors (58).

It is worth mentioning that the COVID-19 pandemic had no serious consequences for exercise interventions and data collection. Exercise interventions could continue while adhering to strict hygiene measures. In particular, at the beginning of the pandemic, which is equivalent to the last third of the study, contact times at outpatient follow-up visits were reduced to a minimum. Following a COVID-19 diagnosis, one participant of the CG was isolated over 14 days. The disease was asymptomatic, and the interventions could be continued after isolation period without any restrictions.

Our hypothesis of the superiority of strength training as the more appropriate exercise method compared with standard care for accomplishing daily tasks was not confirmed in the ActiveADL study. In our cohort, it became clear that the training content may play a subordinate role. Instead, regular exercise sessions to maintain the PA during treatment, as well as individual adaptation of low-to-moderate exercise intensity to the patient's performance and health status, could be essential to support the ability to continue ADL accomplishment. Our results demonstrate that a population of patients with ALL, AML, and NHL can benefit from supervised exercise interventions in the course of acute therapy in general.

With regard to recruitment- and assessment-related limitations, we refer to the publication of the baseline data (27). In the following, we present exercise-specific limitations. Due to the small number of initial diagnoses of childhood, cancer at single sites and combined with the self-determination of children in the treatment process, the definition of a homogeneous study cohort for an RCT is a challenging task. Conclusions regarding intervention effects for other cancer types cannot be made. The small number of participants in each group as well as the skewed gender and age distribution limit further subgroup analysis and generalizability of the results. The findings suggest that a larger sample size is needed to clarify the exercise efficiency of specific training methods. To avoid additional burden on participants due to the number of assessments and activity measurements during the intensive treatment, secondary outcomes were collected at only two visits. To provide stronger conclusions about the effects of exercise on motor performance and PA, the MOON-test and accelerometer should have been used at V3. Strict adherence to visits coupled with the defined therapy phases of treatment protocols could not be consistently maintained. Individual measurement time points were adjusted to ensure the participants' regeneration episodes. Associated with a high total score at V0 and the low possibility of improvement in the course of treatment, Functional ADL Screen results suggest a ceiling effect. According to the three exercise physiologists distributed over two study sites, a standardized training over a 3.5-year study duration was possible only to a limited extent. However, clear documentation, predefinition of a strength training manual and regular agreements, contributed to interventions as standardized as possible during the long study period.

## Conclusion

Our results indicate the relevance of a regular, supervised exercise program throughout the acute anticancer treatment to maintain the children's autonomy and participation in clinical routine and potentially counteract physical inactivity and motor performance impairments. In this context, a specific strength training could not be shown to be the method of first choice, as the outcome parameters of the CG with standard care exercise program have also been stabilized in the course of therapy. However, the ActiveADL Study illustrates that patients should have access to a structured and holistic exercise program early after diagnosis. Considering the complex interplay of neuromotor, musculoskeletal, and cognitive mechanisms involved in locomotion and performance of everyday tasks, patients could potentially benefit from a combination of exercise interventions and skilled physical therapy to address the individual needs. For example, physical therapists could provide supporting intervention sessions that cover neuromotor reeducation and mobility training for vincristine peripheral neuropathy or pain due to osteonecrosis. The present findings may be useful for future multicenter studies in defining standardized training content, duration, and intensity of exercise interventions within a homogeneous pediatric cancer cohort. To verify the suspected exercise effects, we are investigating a control cohort at a study site without an implemented exercise program, who did not receive any exercise interventions, at the cessation of intensive.

## Data Availability

The raw data supporting the conclusions of this article will be made available by the authors, without undue reservation.
